# COMING OUT AND GOING BACK IN: AGING AND STRATEGIC IDENTITY DISCLOSURE AMONG LGBTQ ADULTS

**DOI:** 10.1093/geroni/igac059.1320

**Published:** 2022-12-20

**Authors:** Brian Sweeny

**Affiliations:** Long Island University, Brookville, New York, United States

## Abstract

In addition to facing social, economic, and health disparities, LGBTQ older adults face prejudice and discrimination across various social domains, leading some to re-enter the closet. When might older adults openly identify as LGBTQ, and when might they conceal this identity? Based on participant observation and interview data collected in two naturally occurring retirement communities in New York City, this paper examines LGBTQ identity and coming out in relation to aging processes, especially changing care networks and family structures, decreasing autonomy, and increasing engagement with health and social services. The older LGBTQ adults in the study feel the benefits of broad social changes and increasing cultural competencies regarding LGBTQ issues, yet they also speak of unsafe spaces, experiences of exclusion, and uncertainties. I analyze three specific identity disclosure strategies older LGBTQ adults use as they navigate prejudice and discrimination and work to affirm identities as aging gender and sexual minorities.

